# Evaluating the Potential Impact of AI on Urinary Tract Infection Diagnosis in the Emergency Department Across Demographic Groups: Retrospective Cohort Study

**DOI:** 10.2196/91148

**Published:** 2026-05-06

**Authors:** Mark Iscoe, Huan Li, Haipeng Xue, Vimig Socrates, Aidan Gilson, Thomas Huang, Richard Andrew Taylor

**Affiliations:** 1 Department of Emergency Medicine, School of Medicine, Yale University, 464 Congress Ave # 260, New Haven, CT, 06519, United States, 1 (203) 785-2353; 2 Department of Biomedical Informatics and Data Science, School of Medicine, Yale University, New Haven, CT, United States; 3 School of Medicine, Yale University, New Haven, CT, United States; 4 Program of Computational Biology and Biomedical Informatics, Yale University, New Haven, CT, United States; 5 Department of Opthalmology, Massachusetts Eye and Ear, Harvard University Medical School, Boston, MA, United States; 6 Department of Emergency Medicine, School of Medicine, Johns Hopkins University, Baltimore, MD, United States; 7 Department of Emergency Medicine, School of Medicine, University of Virginia, Charlottesville, VA, United States

**Keywords:** machine learning, emergency medicine, urinary tract infection, artificial intelligence, AI

## Abstract

**Background:**

Urinary tract infection (UTI) is a common emergency department (ED) presentation but can be challenging to diagnose; both overdiagnosis and underdiagnosis are common, and older adults may be at particular risk of misdiagnosis. Artificial intelligence (AI) shows promise in augmenting diagnosis, but performance across patient populations remains underexamined.

**Objective:**

We developed an AI model that combined urine culture positivity prediction and natural language processing (NLP) to predict UTI diagnosis using only information available at the time of a patient’s ED visit. We then evaluated the model’s performance relative to that of physicians in diagnosing UTI across intersectional patient groups.

**Methods:**

We conducted a single-center, multisite retrospective analysis of nonpregnant adult ED patients who had a urinalysis and urine culture test performed during their ED visit at 9 EDs in a single US health system from June 2013 to August 2021. Intersectional groups were defined by binned age (18-44, 45-64, 65-84, and ≥85 years), sex, race, and ethnicity. An Extreme Gradient Boosting classifier model was developed to predict culture positivity (≥10,000 colony-forming units per milliliter) from urinalysis data using 5-fold cross-validation and a 80%-20% train-test split. UTI signs and symptoms were identified using a previously described NLP model. UTI was defined as a positive urine culture and at least 1 UTI sign or symptom identified through NLP. Model performance was evaluated using the area under the receiver operating characteristic curve and rates of overdiagnosis (proportion of patients without UTI mistakenly diagnosed with UTI) and underdiagnosis (proportion of patients with UTI who were not diagnosed ). Model over- and underdiagnosis rates were compared to those of physicians, with physician diagnosis inferred from a composite proxy outcome of either explicit UTI diagnosis or prescription of a relevant antibiotic in the absence of an alternative infectious disease diagnosis. Cross-group performance variance was assessed through the coefficient of variation (CV) for accuracy and diagnostic odds ratio (DOR).

**Results:**

Of 149,449 included encounters, 22,521 (15.1%) had positive cultures and 20,080 (13.4%) met the definition of UTI. Model area under the receiver operating characteristic curve was 0.93 (95% CI 0.93‐0.93). At a diagnostic threshold of 28%, the model had lower rates of overdiagnosis and underdiagnosis than physicians for each intersectional group. The model’s cross-group CV was 0.039 (95% CI 0‐0.36) for accuracy and 0.48 (95% CI 0.14-0.81) for DOR. Physicians’ CV was 0.080 (95% CI 0-0.40) for accuracy and 0.33 (95% CI 0.004-0.66) for DOR.

**Conclusions:**

In this proof-of-concept study, an AI model had lower overdiagnosis and underdiagnosis rates than a proxy for physician diagnosis across intersectional groups, with comparable cross-group variance. While AI has the potential to augment physicians’ diagnostic accuracy, real-world applications should account for the model’s variable performance across patient groups.

## Introduction

A major concern regarding the use of artificial intelligence (AI) in patient care is the potential to introduce, propagate, or even amplify bias and undermine health equity [1,2]. Nonrepresentative training datasets, errant modeling approaches, and poorly chosen outcomes can all lead to inequitable model performance [3-5]. Despite these challenges, AI as a largely deterministic process also holds significant potential to improve the standardization of care and possibly equity as a consequence [6,7]. However, currently, there are few studies that examine the potential impact of AI on reducing practice variation and associated health inequities and even fewer that do this with an intersectional lens [8-10].

The diagnosis and treatment of urinary tract infections (UTIs) is one clinical domain in which widespread practice variation [11-13] and potential disparities in guideline adherence based on race [14] and age [11,14] indicate a potential role for AI to augment clinicians’ decision-making and promote standardized, equitable care. In the United States, there are more than 3 million emergency department (ED) visits each year for UTI [15-17]. UTI diagnosis and management are complicated by the fact that the laboratory gold standard for diagnosis, the urine culture, does not result within the timeframe of an ED visit; ED clinicians must therefore act on incomplete information, and there are high rates of both over- and underdiagnosis [18-20]. Both carry risks for patients: overdiagnosis can lead to extended hospital stays [21]; missed alternative diagnoses [22]; and treatment side effects including *Clostridioides difficile* infections [23] and antibiotic resistance, which has implications for both individual and public health [24,25], and underdiagnosis puts patients at risk of ongoing symptoms as well as UTI complications including sepsis [26], delirium, and falls resulting in injury [27-29]. Misdiagnosis can also increase patient costs [30]. The burden of misdiagnosis is not evenly distributed across patient groups; notably, there are higher rates of overtreatment in older adults [31-33].

Prior work indicates that AI offers improved accuracy as compared to physician judgment in UTI diagnosis [34,35], but it is unclear whether these differences translate to vulnerable subgroups or whether AI might demonstrate less bias across these subgroups than physicians and, therefore, have the potential to improve physician performance by mitigating bias. To address these knowledge gaps, the primary objective of this investigation was to compare physician and AI performance in UTI diagnosis through an intersectional lens.

## Methods

### Design, Study Population, and Setting

We conducted a single-center, multisite retrospective cohort analysis of nonpregnant adult (aged ≥18 years) ED patients who had a urinalysis and urine culture performed during their ED visit and were ultimately discharged from the ED. We excluded pregnant patients because of distinct guideline recommendations on the treatment of asymptomatic bacteriuria in this population [36]. We limited our study to discharged patients as this group represents relatively lower-risk patients less likely to receive empiric antibiotics for undifferentiated suspected infections. Data were collected between June 2013 and August 2021 from 9 EDs in a single northeastern US regional health network.

### Data Collection and Preprocessing

All data for model development and assessment were obtained from the system-wide electronic health record (EHR) data warehouse (Epic). Extracted variables included patient demographics, presenting concerns, ED diagnoses, prescriptions, ED dispositions, urinalysis results, and clinical notes to identify UTI signs and symptoms through natural language processing (NLP) [37]. We included missing values without further imputation. Over the course of the study, various study sites used several different scales and conventions to report components of urinalysis results. We reconciled various ordinal (qualitative and semiquantitative) and quantitative scales into a single ordinal scale for each urinalysis result component.

### Definition of Culture Positivity

We considered a urine culture to be positive if it grew 10,000 or more colony-forming units per milliliter of a pathogenic bacterial organism [38-41] (see the organism list in Multimedia Appendix 1). This threshold is consistent with a recent multidisciplinary Delphi consensus study [39] and is our health system laboratories’ cutoff for reporting organisms in urine cultures.

### Definition of UTI

In each of the analyses described below, we used a “strict” UTI definition that, consistent with society guidelines and interdisciplinary consensus statements [39,42,43], required both culture positivity (see the definition above) and the presence of at least one UTI sign or symptom (see the list in Multimedia Appendix 1) as identified through NLP of the ED clinician’s note from the ED visit in question (see the brief UTI definitions in Table 1). By requiring the presence of a UTI sign or symptom, we distinguished true UTI from asymptomatic bacteriuria, which typically does not require treatment.

**Table 1.** Strict and liberal urinary tract infection (UTI) outcome definitions; details can be found in the main text and Multimedia Appendix 1.

UTI definition	Details
Strict (primary analysis)	Positive urine culture (≥10,000 colony-forming units per milliliter of a pathogenic organism) AND at least one UTI sign or symptom identified through natural language processing
Liberal (sensitivity analysis)	Positive urine culture (based on the definition above)

Using methods previously described [37], we used a transformer-based large language model (Clinical-Longformer [44]) fine-tuned on ED notes to identify signs and symptoms of UTI through the NLP task of named entity recognition. In our prior work, this model showed excellent performance in identifying the presence of any UTI signs or symptoms at the note level, with precision of 0.97 (95% CI 0.93‐0.98), recall of 0.99 (95% CI 0.96-0.99), and *F*_1_-score of 0.98 (95% CI 0.95-1.0) when evaluated in the same setting [37]. The included UTI signs and symptoms were selected based on literature review [15,45,46], society guidelines [42,43], and expert opinion.

As a sensitivity analysis, we also used a “liberal” UTI definition requiring only urine culture positivity (based on the aforementioned definition) using the same urine culture prediction model as that in our “strict” UTI definition (Table 1). This liberal definition was used to account for variances in patient presentations, clinician documentation, and NLP performance and to give clinicians the benefit of the doubt assuming that a urine culture was only ordered if a positive result would warrant treatment.

### Identification of Physician UTI Diagnosis

Because physicians vary in their documentation of visit diagnoses and antibiotics are prescribed for many diagnoses apart from UTIs, we created a composite proxy outcome to capture presumed physician UTI diagnosis. We considered a physician to have diagnosed their patient with a UTI in one of two conditions: (1) the patient was given an explicit diagnosis of a UTI (cystitis, pyelonephritis, unspecified UTI, or a synonym) *or* (2) they received a prescription for an antibiotic that could presumably treat a UTI *and* were given a nonspecific diagnosis that could likely be attributed to a UTI *and* were *not* given an alternative diagnosis that would explain their antibiotic prescription. Lists of included antibiotics, UTI symptoms, and alternative diagnoses were created based on society guidelines [45], published literature [38-41], reference texts [47,48], and expert consensus between study authors (MI and RAT) and can be found in Multimedia Appendix 1.

### Intersectional Groups

We used an intersectional lens [8] to examine equity in UTI diagnosis across patient groups. In defining intersections, patients were grouped by sex [49,50] and age [50,51] based on known epidemiologic risk factors between these groups as well as evidence on varied UTI treatment guideline adherence based on age [11,14] and race and ethnicity given some evidence on disparities in guideline adherence across these axes [14,52]. Age was binned into 4 groups: 18 to 45 years, 45 to 64 years, 65 to 84 years, and 85 years or above. Race and ethnicity were obtained from the EHR database and were based on patients’ self-reported responses to a prompt at health system registration, with options of “Hispanic or Latina/o/x,” “non-Hispanic,” and “unknown” for ethnicity and “American Indian/Alaska Native,” “Asian,” “Black or African American,” “multiracial,” “Native Hawaiian/Pacific Islander,” “other/not listed,” and “White or Caucasian” for race. Sex data were also obtained from the EHR database based on patients’ self-reported responses to a prompt at health system registration.

All patients were included in model development and overall model evaluation. However, in examining model performance across intersectional groups, we limited our analyses to the 19 intersectional groups with at least 2000 patients to ensure adequate sample size for meaningful comparison.

### Predictive Model Development

#### Outcome

We trained a model to predict likelihood of urine culture positivity based on the aforementioned definition. Standard components of our laboratories’ urine dipstick (blood, glucose, ketones, leukocyte esterase, nitrites, and protein) and urine microscopy (bacteria, epithelial cells, and white blood cells) and clinical site were used as predictor variables. This decision was made for several reasons: urinalysis data (we use the term “urinalysis” to collectively refer to urine dipstick and urine microscopy testing) are objective and readily available during an ED visit; urinalysis and urine culture specimens are typically drawn from the same urine sample, and it is logical that the results of the former can predict the latter; exclusion of demographic data avoids bias related to variable UTI rates across groups; and exclusion of past medical history, presenting concern, and other EHR data avoids bias related to variable health care access, record completeness, or documentation. Site was included to account for site-specific variance in urinalysis reporting procedures and assays.

#### Extreme Gradient Boosting Classifier

Our model was based on an Extreme Gradient Boosting (XGBoost) classifier, one of the most widely used ensemble tree-based classification methods [53]. After stratifying the target variables by unique patients, we assigned weights to each observation during training based on the ratio of positive and negative classes to prevent potential bias caused by data imbalance. We used the Optuna [54] package in Python (Python Software Foundation) for hyperparameter tuning, optimizing a customized objective function to maximize the area under the receiver operating characteristic curve (AUROC), and found the best hyperparameters with respect to each target variable. Optuna performed the search by sampling hyperparameters from predefined space using Bayesian optimization and pruning unpromising trials to reduce computation. To avoid potential overfitting during tuning, we used 5-fold cross-validation. The dataset was randomly divided into training (80%) and testing (20%) sets. Hyperparameter tuning was performed on the training set. The optimized model was applied to the held-out testing set to examine performance across candidate thresholds and determine the operating threshold. After fixing the model configuration, 5-fold cross-validation was performed on the full dataset to estimate model performance across the entire cohort. Shapley Additive Explanations (SHAP) values were used to interpret the model’s feature importance and directional impact on the AI model’s behavior [55].

#### Incorporation of NLP

In our primary (“strict”) UTI definition, the model’s final UTI diagnosis was based on XGBoost classifier algorithm predictions of urine culture positivity and the presence of at least one UTI sign or symptom as identified through NLP of the ED clinician’s initial note. The sign or symptom requirement was applied as a binary rule (rather than a probabilistic prediction). In contrast, for our sensitivity analyses using the “liberal” UTI definition, which did not require signs or symptoms of UTI to make the UTI diagnosis, we did not incorporate NLP and simply based diagnosis on the XGBoost model’s predictions.

### Outcomes

#### Overall Model Performance

We assessed model performance using standard predictive model evaluation metrics, including ROC-AUC, area under the precision-recall (PR) curve, and calibration curves.

#### Comparison of Model vs Physician Equity Across Intersections

To examine performance and equity across intersectional groups, we calculated and compared model and physician over- and underdiagnosis rates for each intersectional group meeting our sample size threshold. This involved quantifying the instances in which UTIs were diagnosed in excess (overdiagnosis or false-positive rate, defined as the proportion of total negative cases misidentified as positive) or were missed (underdiagnosis or false-negative rate, defined as the proportion of total positive cases misidentified as negative).

#### Selection of Decision Threshold

We analyzed model performance at 3 different decision thresholds (ie, the threshold of model-predicted culture positivity probability at which a case was classified as positive). For our primary analysis, we selected a policy-constrained operating point designed to maintain a false-negative rate below that of physicians for all intersectional groups, thereby establishing a threshold at which no intersectional group would have an increase in missed diagnoses in a theoretical scenario in which the model was applied in place of human judgment; this threshold was selected as an analytic exercise rather than a policy recommendation. To identify this threshold, we decreased the decision threshold stepwise by 1 percentage point at a time and compared the model’s false-negative rate according to the strict definition to physicians’ false-negative rate for each intersectional group.

As a sensitivity analysis, we tested model performance at 2 additional thresholds. First, we examined a treatment threshold of 42.3%, which was previously reported as the probability threshold at which 50% of primary care clinicians would treat for UTI [56]. Second, we evaluated model performance at the “optimal” decision threshold, defined as the threshold at which the difference between true-positive rate and false-positive rate was maximized [57]; it should be noted that, depending on how the relative risks and benefits of appropriate treatment, overtreatment, and undertreatment are weighed, this statistically optimal threshold may not be clinically optimal. We also indirectly estimated physicians’ decision threshold by calculating physician diagnosis rates for each decile of model-predicted likelihood of urine culture positivity.

#### Comparison of Model and Physician Coefficient of Variation

Our primary outcome for determining diagnostic equity across intersectional groups was the coefficient of variation (CV; the ratio of SD to the mean) [58] across intersectional groups for 2 balanced classification metrics: accuracy (proportion of correct predictions) and diagnostic odds ratio (DOR; the ratio of the odds of a positive test in patients with the disease to the odds of a positive test in patients without the disease) [59]. We selected balanced metrics (ie, those that account for errors of over- or underdiagnosis) over metrics such as statistical parity difference and equal opportunity difference that only account for errors in one direction because of the risks associated with both over- and underdiagnosis of UTI. The 95% CIs of the CV estimates were calculated via normal approximation [60].

#### Analyzing Key Drivers and Influential Variables

We identified the top variables influencing model classification using SHAP values [55].

### Patient and Public Involvement

Patients were not directly involved in research question or outcome measure development.

### Ethical Considerations

The Yale School of Medicine Institutional Review Board approved this research and waived the need for informed consent (1602017249). All data was deidentified prior to analysis and stored on secure servers to maintain confidentiality. Participants were not compensated.

## Results

### Overview

A total of 149,449 encounters met the inclusion criteria, of which 22,521 (15.1%) had a positive urine culture, meeting our liberal UTI definition; of these, 20,080 (89.2%; 20,080/149,449, 13.4% of total cases) had at least one UTI sign or symptom as identified through NLP, meeting our strict UTI definition. Median age was 50 years (IQR 31-67); 71.2% (106,380/149,449) of the patients were female; 56.2% (84,005/149,449) were White individuals; 22.1% (33,070/149,449) were Black individuals; and 25.6% (38,231/149,449) were Hispanic or Latina, Latino, or Latinx. Full baseline patient characteristics, UTI symptoms as identified through NLP, and urine culture results are shown in Table 2. Model feature completeness and missingness are provided in Multimedia Appendix 1.

**Table 2.** Baseline emergency department encounter and patient characteristics (N=149,449).

Variable	UTI^a^ (symptoms and positive culture; n=20,080), n (%)	No UTI (n=129,369), n (%)	Overall, n (%)
Sex			
Female	16,186 (80.6)	90,194 (69.7)	106,380 (71.2)
Male	3894 (19.4)	39,175 (30.3)	43,069 (28.8)
Age group (y)			
18-44	8043 (40.1)	61,174 (47.3)	69,217 (46.3)
45-64	4614 (23)	33,722 (26.1)	38,336 (25.7)
65-84	4764 (23.7)	24,617 (19)	29,381 (19.7)
≥85	2659 (13.2)	9856 (7.6)	12,515 (8.4)
Self-reported race			
American Indian or Alaska Native	47 (0.2)	441 (0.3)	488 (0.3)
Asian	295 (1.5)	1980 (1.5)	2275 (1.5)
Black or African American	3932 (19.6)	29,138 (22.5)	33,070 (22.1)
Native Hawaiian or Pacific Islander	61 (0.3)	441 (0.3)	475 (0.3)
White	11,659 (58.1)	72,346 (55.9)	84,005 (56.2)
Other or not listed	3877 (19.3)	23,758 (18.4)	27,604 (18.5)
Unknown or patient declined	209 (1)	1292 (1)	1501 (1)
Self-reported ethnicity			
Hispanic or Latina, Latino, or Latinx	5239 (26.1)	32,992 (25.5)	38,231 (25.6)
Non-Hispanic or Latina, Latino, or Latinx	14,738 (73.4)	95,707 (74)	110,445 (73.9)
Unknown	103 (0.5)	670 (0.5)	773 (0.5)
Preferred language			
English	17,308 (86.2)	113,336 (87.6)	130,644 (87.4)
Other	2772 (13.8)	16,033 (12.4)	18,805 (12.6)
Past medical history			
Prior UTI	15,746 (78.4)	42,453 (32.8)	58,199 (38.9)
Diabetes	2819 (14)	14,992 (11.6)	17,811 (11.9)
Dementia	2347 (11.7)	10,301 (8)	12,648 (8.5)
UTI signs and symptoms (identified through NLP^b^)^c^			
Likely UTI symptom	17,253 (85.9)	94,267 (72.9)	111,520 (74.6)
Likely UTI examination finding	4920 (24.5)	19,649 (15.2)	24,569 (16.4)
Systemic symptom potentially attributed to UTI	10,025 (49.9)	53,319 (41.2)	63,344 (42.4)
Any sign or symptom	20,080 (100)	111,633 (86.3)	131,713 (88.1)
UTI diagnosis or presumed diagnosis (clinician decision)	12,735 (63.4)	11,807 (9.1)	24,542 (16.4)
Culture positivity	20,080 (100)	2441 (1.9)	22,521 (15.1)

a UTI: urinary tract infection.

b NLP: natural language processing.

c Details of symptom identification and classification are provided in Multimedia Appendix 1.

### Intersectional Groups

**Figure 1.**
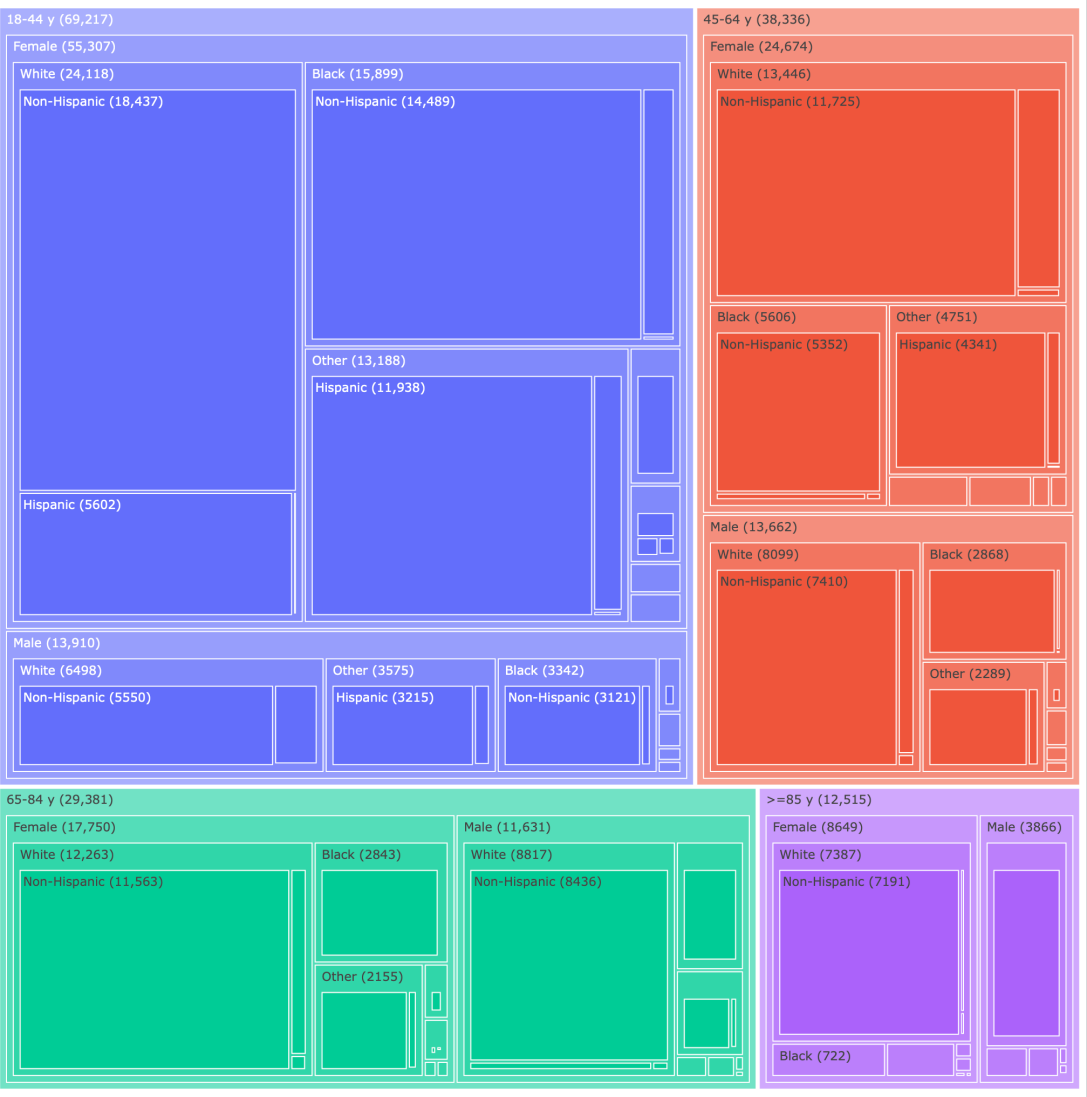
Tree map of intersectional groups nested by categories (in order) of age bin, sex, race, and ethnicity. Box size corresponds to patient count, shown in parentheses. To improve figure readability, the racial category “Black or African American” was abbreviated as “Black,” and the ethnic category “Hispanic or Latina, Latino, or Latinx” was abbreviated as “Hispanic” in figure labels. Labels were omitted for boxes that were too small to label legibly.

**Figure 2.**
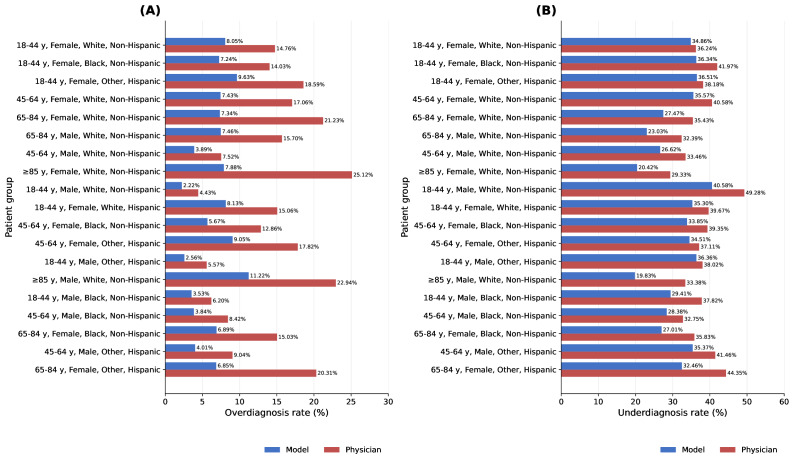
Model and physician performance according to our strict urinary tract infection definition: (A) overdiagnosis and (B) underdiagnosis. Model predictions are shown at a diagnostic threshold of 28%. To improve figure readability, the racial category “Black or African American” was abbreviated as “Black,” and the ethnic category “Hispanic or Latina, Latino, or Latinx” was abbreviated as “Hispanic” in figure labels.

A total of 19 intersectional groups of age bracket, sex, race, and ethnicity met our threshold of 2000 encounters (see the tree map in Figure 1). Physician and model diagnostic performance by group is shown in Figure 2.

### Overall Physician Performance

A UTI diagnosis was made in 63.4% (12,735/20,080) of total cases with a UTI. Physicians had an overall false-positive rate of 9.1% (11,807/129,369) and an overall false-negative rate of 36.6% (7345/20,080).

### Overall Predictive Model Performance

**Figure 3.**
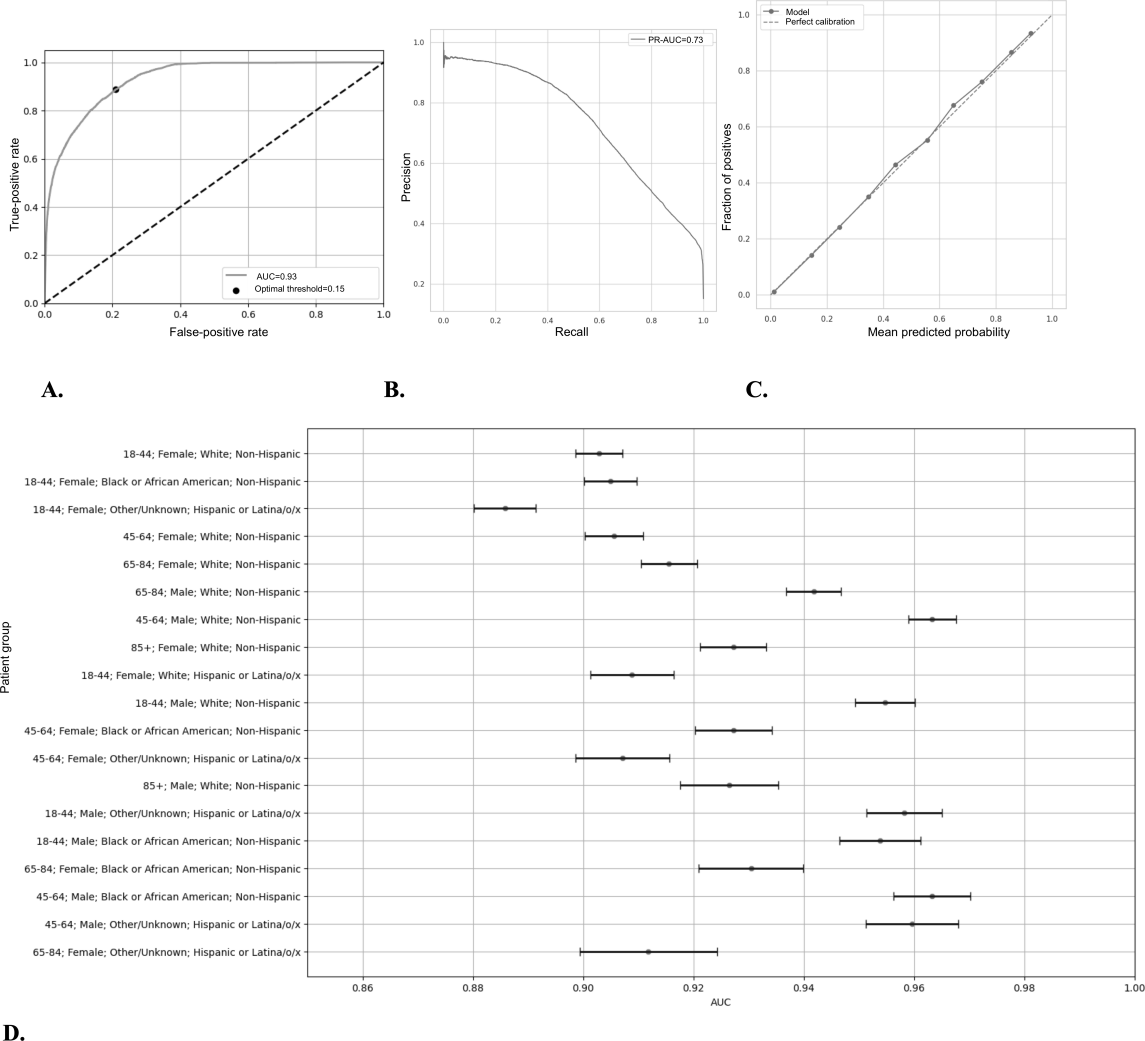
Extreme Gradient Boosting model performance in predicting urine culture positivity: (A) model receiver operating characteristic (ROC) curve, (B) precision-recall curve, (C) calibration curve, and (D) AUC by intersectional group with 95% CIs. AUC: area under the receiver operating characteristic curve.

The XGBoost classifier model achieved an ROC-AUC of 0.93 (95% CI 0.93-0.93). The area under the PR curve was 0.74 (95% CI 0.74-0.74). The receiver operating characteristic curve, PR curve, calibration curve, and ROC-AUC by intersectional group are shown in Figure 3. Model performance across intersectional groups using the decision threshold of 28% predicted the probability of a positive culture, the highest integer threshold at which the model’s false-negative rate for each intersectional group was lower than that of physicians (see the Selection of Decision Threshold section), is shown in Figure 2. At this decision threshold, the model also had a lower false-positive rate than physicians for each intersectional group; overall model false-positive rate at this threshold was 7.8% (95% CI 7.6%-7.9%). and the false-negative rate was 32% (95% CI 30.9%-32.1%). Overall model accuracy was 0.89, and DOR was 25.8. The “optimal” decision threshold based on the aforementioned definition was identified at 15%. Model performance according to the “liberal” UTI definition (requiring culture positivity alone) and at other decision thresholds can be found in Multimedia Appendix 1. Multimedia Appendix 1 also shows rates of culture positivity for each decile of model-predicted likelihood.

### Variation Across Groups

The CV for model performance across intersectional groups at a decision threshold of 28% was 0.039 (95% CI 0-0.36) for accuracy and 0.48 (95% CI 0.14-0.81) for DOR. Physicians had a CV of 0.080 (95% CI 0-0.40) for accuracy and 0.33 (95% CI 0.004-0.66) for DOR.

### Key Drivers and Influential Variables

**Figure 4.**
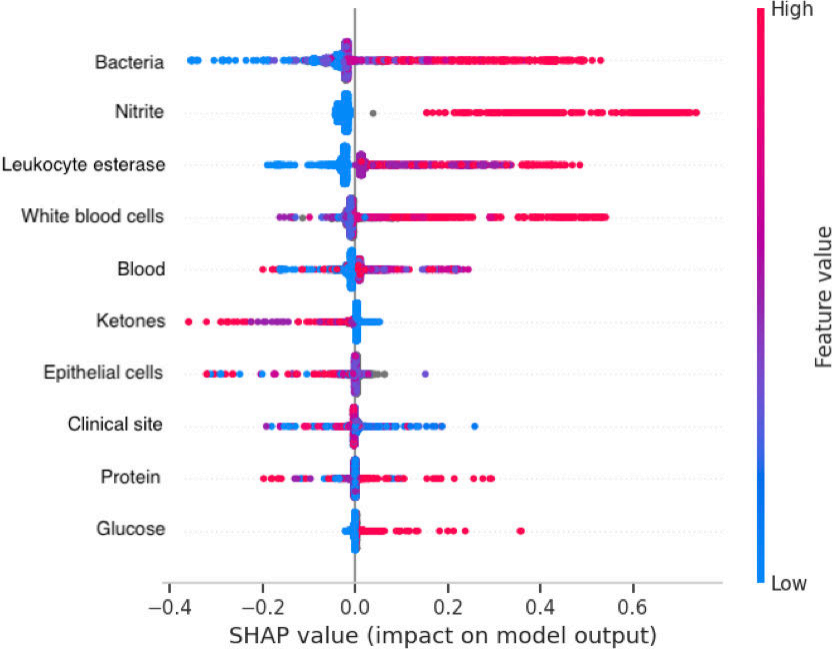
Shapley Additive Explanations (SHAP) values showing Extreme Gradient Boosting model feature importance. Color indicates feature value, as indicated on the right-sided y-axis; placement along the x-axis indicates positive (right) or negative (left) impact on model prediction. For example, we see that the presence of nitrite has a strong positive impact on model prediction but the absence of nitrite has only a slight negative impact on model prediction.

A SHAP value waterfall plot depicting the features’ importance on model predictions and the directionality of that impact is shown in Figure 4.

## Discussion

### Principal Findings

In this retrospective observational study of nonpregnant adult patients discharged from the ED, we found that an XGBoost machine learning model trained solely on urinalysis results and the clinical site performing the urinalysis had excellent performance in predicting urine culture positivity. When combined with NLP to detect UTI signs and symptoms, the model had lower over- and underdiagnosis rates than our composite proxy for physicians’ real-world UTI diagnoses for each intersectional group defined by age, sex, race, and ethnicity, with similar variation in performance across groups. These findings suggest that an AI model has the potential to augment ED care by decreasing both over- and underdiagnosis of UTI with comparable variation in diagnostic equity to that of current physician performance. Our predictive model performed at the high end of the range reported in a recent meta-analysis of 14 UTI prediction models, which found a pooled area under the curve of 0.89 (95% CI 0.86-0.92) [61], and to our knowledge, our study is the first to report performance across intersectional groups.

In contrast to some prior examples of instances in which AI has been shown to introduce or propagate bias, we suspect that our model had a relatively stable performance across groups because (1) it was trained on an objective gold standard (ie, a laboratory result rather than one involving physician judgment) and (2) the most impactful predictors as indicated by SHAP values—urinalysis bacteria, nitrite, leukocyte esterase, and white blood cells—were not only objective but also biologically related to the outcome of interest. In a widely cited 2019 study by Obermeyer et al [3], a commercial algorithm used to identify patients with complex health needs was found to exhibit substantial racial bias, largely because the model was trained using health costs as a proxy for health need and costs are not equitable across demographic groups. In contrast, this study used a direct, objective outcome (urine culture results) rather than a proxy, reducing the risk of bias. A 2022 study by Juhn et al [62] examining the performance of a machine learning model to predict pediatric asthma exacerbations found that the model performed worse in children with lower socioeconomic status, which the authors postulated was due to greater missingness in EHR data pertaining to past medical history. In a 2023 study examining the effects of simulated missing data in a cohort of intensive care unit patients, Getzen et al [63] found that missingness more negatively impacted disease prediction model performance in groups with less health care access. Because the various components of the urinalysis are routinely performed and reported together, there was relatively low missingness in our study and no reason to suspect inequitable distribution of missing data based on health care access or other factors, decreasing the risk of missingness bias.

While our model performed strongly in all intersectional groups, similar to physicians, it did not perform *equally* across groups. In particular, we noted that overdiagnosis was more common in older adult patients; for both the model and physicians, the highest rates of overdiagnosis were observed in male and female White, non-Hispanic or Latina, Latino, or Latinx patients aged 85 years or older, the only 2 groups in this age bracket who met the sample size threshold for inclusion in the intersectional analysis; these 2 groups also had the lowest rates of underdiagnosis for both physicians and the model. Conversely, the 3 groups with the lowest rates of overdiagnosis for both physicians and the model comprised male individuals aged 18 to 44 years. Potential explanations for these age- and sex-related disparities include baseline differences in UTI rates; difficulty obtaining a clean-catch specimen in older adults [16,64]; varying rates of bacterial colonization [31,65]; and, in the case of physician decision-making, diagnostic biases and varying risk-benefit calculations guiding testing and treatment thresholds. Prior literature has similarly demonstrated decreased model performance in older adult populations in other domains [66]. Future work should explore the creation of a predictive model specific to this high-risk group.

In our sensitivity analysis using a “liberal” UTI definition that relied solely on urine culture results and did not require the presence of UTI signs or symptoms, the model also had lower over- and underdiagnosis rates when compared to the proxy for physician diagnosis for each intersectional group, although the difference between model and physician overdiagnosis rates was less pronounced than for the strict UTI definition. This finding illustrates that the model’s strong performance relative to physicians was driven not just by physicians’ misdiagnosis of asymptomatic bacteriuria as UTI but also by physicians’ incorrect predictions of urine culture positivity. By removing the requirement for UTI signs or symptoms, we also eliminated the possibility that our analysis incorrectly classified patients as not having had a UTI due to NLP errors, incomplete documentation, or case-specific clinical nuances. The decision to include or exclude the NLP component of the model in future real-world applications would depend on the use case. We believe that NLP is valuable for disease and treatment surveillance as it can help establish the gold-standard diagnosis; however, its utility may be lower in real-time clinical decision support as clinicians are likely already familiar with the details of their patients’ presentations and the documentation required for NLP often lags behind clinical decision-making and diagnosis. Looking ahead, as ambient technologies are increasingly used in clinical care, real-time incorporation of clinical data may become more feasible.

A novel observation in this study is that variation in the model’s diagnostic performance across intersectional groups differed depending on both the decision threshold selected and the test characteristic evaluated such that, for some test characteristics and decision thresholds, the model demonstrated less cross-group variance than physicians and, for others, it demonstrated increased variance (Multimedia Appendix 1). These findings suggest that evaluations of algorithmic performance and equity should carefully consider the clinical situation in which a model will be applied, taking into account anticipated decision thresholds and which test characteristics to prioritize, among other factors. This calculation requires an understanding of the relative risks and benefits associated with correct classification and misclassification for positive and negative cases and may vary by patient and scenario, often including information that is not captured in the EHR.

From the standpoint of resource use, our model’s performance suggests that one potential application could be avoidance of urine culture testing in very low-risk patients. Of note, 60% (89,741/149,449) of patients had predicted likelihoods of urine culture positivity below 10%; in this population, the rate of urine culture positivity was 1.1% (979/89,741), which is likely below the threshold for testing for most clinicians in many clinical scenarios. Future applications could consider using AI to help guide decision-making on urine culture testing, potentially reducing low-value testing.

### Limitations

Our study has several limitations. First, we should note that the decision threshold we analyzed was a policy-constrained threshold that does not reflect an analysis of the relative benefits and risks associated with appropriate diagnosis and treatment, overdiagnosis, and missed or delayed diagnosis. The ideal threshold likely varies with clinical and patient factors, and future work could explore decision analysis to better guide population- and patient-level care.

Other limitations relate to the generalizability of our findings. While we included data from 9 EDs over nearly a decade, all sites were part of a single regional health network, and our findings, particularly regarding physician practice patterns, may not be applicable to all care settings; external validation would be necessary before broader implementation. An additional limitation to generalizability is that we only studied discharged patients with a urine culture ordered. Performance in admitted patients has not yet been evaluated, and clinicians may have different thresholds for treating these patients as they await urine culture results. We cannot directly comment on performance in patients who had a urinalysis but no urine culture as they lack outcome data.

Three additional limitations arise from the challenge of retrospectively identifying diagnoses from EHR data. First, in certain situations, clinicians may have identified and acknowledged the possibility of a UTI but elected to defer treatment (and formal diagnosis) until they had the results of the urine culture due to patient-specific risk-benefit considerations, such as concern regarding overprescription of antibiotics and associated downstream harms. Second, because physicians do not always explicitly code their UTI diagnoses, we developed a composite proxy outcome of explicit or presumed diagnosis, as detailed in the Methods section; while the large majority of patients diagnosed with UTI (22,574/24,542, 92%) had an explicit UTI diagnosis, some of those with a presumed diagnosis may have been misclassified if they were in fact prescribed antibiotics for an alternative diagnosis that was not explicitly made or was not among the alternative diagnoses that we included (Multimedia Appendix 1). Third, as discussed above, the “strict” UTI definition used in our primary analysis relied on NLP to identify signs and symptoms of UTI; while the model we used showed excellent performance in our prior evaluation [40], it may have misclassified some cases, including ones in which patients with UTIs had atypical symptoms or, conversely, had symptoms potentially attributable to UTI that had an obvious alternative etiology (eg, abdominal pain due to trauma). However, as noted above, the model also performed strongly in our sensitivity analysis using a “liberal” UTI definition that did not involve NLP.

### Conclusions

This proof-of-concept study demonstrates that an AI algorithm trained on a small set of objective features that do not include patient demographics can have consistently strong performance across demographic intersectional groups and has the potential to augment clinicians’ decision-making. Nonetheless, performance varied across groups, and any real-world application should take that variance into account. Moreover, appropriate clinical application of AI in UTI diagnosis requires consideration of the case-specific diagnostic probability threshold at which treatment is warranted, as well as the presence or absence of clinical signs or symptoms that distinguish UTI from asymptomatic bacteriuria.

## Supplementary material

10.2196/91148 Multimedia Appendix 1 Additional details on methods and secondary analyses.

## Abbreviations

AI: artificial intelligence
CV: coefficient of variation
DOR: diagnostic odds ratio
ED: emergency department
EHR: electronic health record
NLP: natural language processing
PR: precision-recall
ROC-AUC: area under the receiver operating characteristic curve
SHAP: Shapley Additive Explanations
UTI: urinary tract infection
XGB: Extreme Gradient Boosting

## References

[1] Challen R, Denny J, Pitt M, Gompels L, Edwards T, Tsaneva-Atanasova K (2019). Artificial intelligence, bias and clinical safety. BMJ Qual Saf.

[2] Celi LA, Cellini J, Charpignon ML (2022). Sources of bias in artificial intelligence that perpetuate healthcare disparities-a global review. PLOS Digit Health.

[3] Obermeyer Z, Powers B, Vogeli C, Mullainathan S (2019). Dissecting racial bias in an algorithm used to manage the health of populations. Science.

[4] Mittermaier M, Raza MM, Kvedar JC (2023). Bias in AI-based models for medical applications: challenges and mitigation strategies. NPJ Digit Med.

[5] Seyyed-Kalantari L, Zhang H, McDermott MB, Chen IY, Ghassemi M (2021). Underdiagnosis bias of artificial intelligence algorithms applied to chest radiographs in under-served patient populations. Nat Med.

[6] Kelly CJ, Karthikesalingam A, Suleyman M, Corrado G, King D (2019). Key challenges for delivering clinical impact with artificial intelligence. BMC Med.

[7] Celi LA, Hinske C, Alterovitz G (2008). Artificial intelligence to reduce practice variation in the ICU. Crit Care.

[8] Samra R, Hankivsky O (2021). Adopting an intersectionality framework to address power and equity in medicine. Lancet.

[9] Bauer GR, Lizotte DJ (2021). Artificial intelligence, intersectionality, and the future of public health. Am J Public Health.

[10] Lee MS, Guo LN, Nambudiri VE (2022). Towards gender equity in artificial intelligence and machine learning applications in dermatology. J Am Med Inform Assoc.

[11] Langner JL, Chiang KF, Stafford RS (2021). Current prescribing practices and guideline concordance for the treatment of uncomplicated urinary tract infections in women. Am J Obstet Gynecol.

[12] Wigton RS, Longenecker JC, Bryan TJ, Parenti C, Flach SD, Tape TG (1999). Variation by specialty in the treatment of urinary tract infection in women. J Gen Intern Med.

[13] Clark AW, Durkin MJ, Olsen MA (2021). Rural-urban differences in antibiotic prescribing for uncomplicated urinary tract infection. Infect Control Hosp Epidemiol.

[14] Kikuchi JY, Banaag A, Koehlmoos TP (2022). Antibiotic prescribing patterns and guideline concordance for uncomplicated urinary tract infections among adult women in the US Military Health System. JAMA Netw Open.

[15] Foxman B (2014). Urinary tract infection syndromes: occurrence, recurrence, bacteriology, risk factors, and disease burden. Infect Dis Clin North Am.

[16] Gordon LB, Waxman MJ, Ragsdale L, Mermel LA (2013). Overtreatment of presumed urinary tract infection in older women presenting to the emergency department. J Am Geriatr Soc.

[17] Ftp.cdc.gov - /pub/health_statistics/NCHS/datasets/NHAMCS/. Centers for Disease Control and Prevention.

[18] Kiyatkin D, Bessman E, McKenzie R (2016). Impact of antibiotic choices made in the emergency department on appropriateness of antibiotic treatment of urinary tract infections in hospitalized patients. J Hosp Med.

[19] Shallcross LJ, Rockenschaub P, McNulty D, Freemantle N, Hayward A, Gill MJ (2020). Diagnostic uncertainty and urinary tract infection in the emergency department: a cohort study from a UK hospital. BMC Emerg Med.

[20] Caterino JM, Leininger R, Kline DM (2017). Accuracy of current diagnostic criteria for acute bacterial infection in older adults in the emergency department. J Am Geriatr Soc.

[21] Petty LA, Vaughn VM, Flanders SA (2019). Risk factors and outcomes associated with treatment of asymptomatic bacteriuria in hospitalized patients. JAMA Intern Med.

[22] Tomas ME, Getman D, Donskey CJ, Hecker MT (2015). Overdiagnosis of urinary tract infection and underdiagnosis of sexually transmitted infection in adult women presenting to an emergency department. J Clin Microbiol.

[23] Petty LA, Vaughn VM, Flanders SA (2020). Assessment of testing and treatment of asymptomatic bacteriuria initiated in the emergency department. Open Forum Infect Dis.

[24] Waller TA, Pantin SA, Yenior AL, Pujalte GG (2018). Urinary tract infection antibiotic resistance in the United States. Prim Care.

[25] Sher EK, Džidić-Krivić A, Sesar A (2024). Current state and novel outlook on prevention and treatment of rising antibiotic resistance in urinary tract infections. Pharmacol Ther.

[26] Kennedy JL, Haberling DL, Huang CC (2019). Infectious disease hospitalizations: United States, 2001 to 2014. Chest.

[27] Soliman Y, Meyer R, Baum N (2016). Falls in the elderly secondary to urinary symptoms. Rev Urol.

[28] Woodford HJ, George J (2009). Diagnosis and management of urinary tract infection in hospitalized older people. J Am Geriatr Soc.

[29] Mayne S, Bowden A, Sundvall PD, Gunnarsson R (2019). The scientific evidence for a potential link between confusion and urinary tract infection in the elderly is still confusing - a systematic literature review. BMC Geriatr.

[30] Shafrin J, Marijam A, Joshi AV (2022). Impact of suboptimal or inappropriate treatment on healthcare resource use and cost among patients with uncomplicated urinary tract infection: an analysis of integrated delivery network electronic health records. Antimicrob Resist Infect Control.

[31] Mody L, Juthani-Mehta M (2014). Urinary tract infections in older women: a clinical review. JAMA.

[32] Middelkoop SJ, van Pelt LJ, Kampinga GA, Ter Maaten JC, Stegeman CA (2021). Influence of gender on the performance of urine dipstick and automated urinalysis in the diagnosis of urinary tract infections at the emergency department. Eur J Intern Med.

[33] Lui S, Carr F, Gibson W (2024). Diagnosis of urinary tract infections in the hospitalized older adult population in Alberta. PLoS One.

[34] Taylor RA, Moore CL, Cheung KH, Brandt C (2018). Predicting urinary tract infections in the emergency department with machine learning. PLoS One.

[35] Burton RJ, Albur M, Eberl M, Cuff SM (2019). Using artificial intelligence to reduce diagnostic workload without compromising detection of urinary tract infections. BMC Med Inform Decis Mak.

[36] U.S. Preventive Services Task Force (2008). Screening for asymptomatic bacteriuria in adults: U.S. Preventive Services Task Force reaffirmation recommendation statement. Ann Intern Med.

[37] Iscoe M, Socrates V, Gilson A (2024). Identifying signs and symptoms of urinary tract infection from emergency department clinical notes using large language models. Acad Emerg Med.

[38] Flores-Mireles AL, Walker JN, Caparon M, Hultgren SJ (2015). Urinary tract infections: epidemiology, mechanisms of infection and treatment options. Nat Rev Microbiol.

[39] Bilsen MP, Conroy SP, Schneeberger C (2024). A reference standard for urinary tract infection research: a multidisciplinary Delphi consensus study. Lancet Infect Dis.

[40] Ronald A (2003). The etiology of urinary tract infection: traditional and emerging pathogens. Dis Mon.

[41] Wagenlehner FM, Bjerklund Johansen TE, Cai T (2020). Epidemiology, definition and treatment of complicated urinary tract infections. Nat Rev Urol.

[42] Loeb M, Bentley DW, Bradley S (2001). Development of minimum criteria for the initiation of antibiotics in residents of long-term-care facilities: results of a consensus conference. Infect Control Hosp Epidemiol.

[43] Nicolle LE, Gupta K, Bradley SF (2019). Clinical practice guideline for the management of asymptomatic bacteriuria: 2019 update by the Infectious Diseases Society of America. Clin Infect Dis.

[44] Li Y, Wehbe RM, Ahmad FS, Wang H, Luo Y (2023). A comparative study of pretrained language models for long clinical text. J Am Med Inform Assoc.

[45] Gupta K, Hooton TM, Naber KG (2011). International clinical practice guidelines for the treatment of acute uncomplicated cystitis and pyelonephritis in women: a 2010 update by the Infectious Diseases Society of America and the European Society for Microbiology and Infectious Diseases. Clin Infect Dis.

[46] Gupta K, Grigoryan L, Trautner B (2017). Urinary tract infection. Ann Intern Med.

[47] Tintinalli JE, John Ma O, Yealy DM (2019). Tintinalli’s Emergency Medicine: A Comprehensive Study Guide.

[48] WikEM.

[49] Griebling TL (2005). Urologic diseases in America project: trends in resource use for urinary tract infections in men. J Urol.

[50] Foxman B (2010). The epidemiology of urinary tract infection. Nat Rev Urol.

[51] Medina M, Castillo-Pino E (2019). An introduction to the epidemiology and burden of urinary tract infections. Ther Adv Urol.

[52] Ramgopal S, Tidwell N, Shaikh N, Shope TR, Macy ML (2022). Racial differences in urine testing of febrile young children presenting to pediatric hospitals. J Racial Ethn Health Disparities.

[53] Chen T, Guestrin C XGBoost: a scalable tree boosting system.

[54] Akiba T, Sano S, Yanase T, Ohta T, Koyama M Optuna: a next-generation hyperparameter optimization framework.

[55] Lundberg SM, Lee SI A unified approach to interpreting model predictions.

[56] Harris A, Pineles L, Baghdadiv JD (2023). Clinician testing and treatment thresholds for management of urinary tract infection. Open Forum Infect Dis.

[57] Bella A, Ferri C, Hernandez-Orallo J, Ramirez-Quintana MJ Quantification via probability estimators.

[58] Abdi H, Salkind N (2010). Encyclopedia of Research Design.

[59] Glas AS, Lijmer JG, Prins MH, Bonsel GJ, Bossuyt PM (2003). The diagnostic odds ratio: a single indicator of test performance. J Clin Epidemiol.

[60] Brown LD, Cai TT, Dasgupta A (2001). Interval estimation for a binomial proportion. Stat Sci.

[61] Shen L, An J, Wang N, Wu J, Yao J, Gao Y (2024). Artificial intelligence and machine learning applications in urinary tract infections identification and prediction: a systematic review and meta-analysis. World J Urol.

[62] Juhn YJ, Ryu E, Wi CI (2022). Assessing socioeconomic bias in machine learning algorithms in health care: a case study of the HOUSES index. J Am Med Inform Assoc.

[63] Getzen E, Ungar L, Mowery D, Jiang X, Long Q (2023). Mining for equitable health: assessing the impact of missing data in electronic health records. J Biomed Inform.

[64] Pallin DJ, Ronan C, Montazeri K (2014). Urinalysis in acute care of adults: pitfalls in testing and interpreting results. Open Forum Infect Dis.

[65] Nicolle LE, SHEA Long-Term-Care-Committee (2001). Urinary tract infections in long-term-care facilities. Infect Control Hosp Epidemiol.

[66] de Groot B, Stolwijk F, Warmerdam M (2017). The most commonly used disease severity scores are inappropriate for risk stratification of older emergency department sepsis patients: an observational multi-centre study. Scand J Trauma Resusc Emerg Med.

