# Supplementary material for: Evaluating the Potential Impact of AI on Urinary Tract Infection Diagnosis in the Emergency Department Across Demographic Groups: Retrospective Cohort Study
Source: JMIR AI. 2026 May 6;5:e91148. doi: 10.2196/91148 (PMC13148603; doi:10.2196/91148)

**SUPPLEMENTAL MATERIALS**

**Supplement 1. List of pathogenic organisms used for culture positivity definition:**

| Achromobacter |
| --- |
| Acinetobacter |
| Actinobaculum |
| Aerococcus |
| Aeromonas |
| Citrobacter |
| Corynebacterium |
| Diphtheroid |
| Enterobacter |
| Enterococcus |
| Escheria  coli |
| Gardnerella |
| Klebsiella |
| Morganella |
| Pantoea |
| Proteus |
| Providencia |
| Pseudomonas |
| Raoultella |
| Salmonella |
| Serratia |
| Shigella |
| Staphylococcus |
| Streptococcus |

**Supplement 2a. List of antibiotics potentially used to treat UTI used for UTI diagnosis definition**

| Amoxicillin |
| --- |
| Amoxicillin |
| Amoxicillin-clavulanate |
| Cefdinir |
| Cefdinir |
| Cefpodoxime |
| Cefuroxime |
| Cephalexin |
| Ciprofloxacin |
| Fosfomycin |
| Levofloxacin |
| Nitrofurantoin |
| Trimethoprim-Sulfamethoxazole |

**Supplement 2b. Diagnoses related to UTI symptoms used for UTI diagnosis definition:**

| Bacteriuria |
| --- |
| Bladder pain |
| Burning with urination |
| Difficulty urinating |
| Dysuria  Flank pain |
| Frequent urination |
| Hematuria |
| Pain with urination |
| Painful urination |
| Pelvic pain |
| Pelvic pressure |
| Pyuria |
| Suprapubic abdominal pain |
| Suprapubic pain |
| Suprapubic pressure |
| Urinary frequency |
| Urinary retention |
| Urinary urgency |

**Supplement 2c. UTI signs/symptom diagnoses used for UTI diagnosis definition:**

| Bacteriuria |
| --- |
| Bladder pain |
| Burning with urination |
| Difficulty urinating |
| Dysuria |
| Flank pain |
| Frequent urination |
| Hematuria |
| Pain with urination |
| Painful urination |
| Pelvic pain |
| Pelvic pressure |
| Pyuria |
| Suprapubic abdominal pain |
| Suprapubic pain |
| Suprapubic pressure |
| Urinary frequency |
| Urinary retention |
| Urinary urgency |

**Supplement 2d. Alternative infectious diagnoses used for UTI diagnosis definition:**

| Abscess |
| --- |
| Appendicitis |
| Bacterial infection |
| Balanitis |
| Balanoposthitis |
| Bite |
| Bursitis |
| Cellulitis |
| Chlamydia |
| Cholangitis |
| Cholecystitis |
| Colitis |
| Dental infection |
| Diarrhea |
| Diverticulitis |
| Endocarditis |
| Endometritis |
| Enteritis |
| Epididymitis |
| Erysipelas |
| Folliculitis |
| Gangrene |
| Gastroenteritis |
| Gingivitis |
| Gonorrhea |
| H. Pylori |
| Laceration |
| Lyme disease |
| Lymphadenitis |
| Lymphangitis |
| Mastitis |
| Meningitis |
| Mycobacterium |
| Neutropenic fever |
| Orchitis |
| Osteitis |
| Osteomyelitis |
| Otitis |
| Paronychia |
| Parotitis |
| Pelvic inflammatory disease |
| Pericoronitis |
| Periodontitis |
| Pharyngitis |
| Phelbitis |
| Pneumonia |
| Pneumonitis |
| Prostatitis |
| Postoperative infection |
| Prophylaxis |
| Pustular infection |
| Pyomyositis |
| Scratch |
| Sexually transmitted disease |
| Sialadenitis |
| Sinusitis |
| Strep throat |
| Tonsillitis |
| Typhlitis |
| Vaginosis |
| Wound infection |

**Supplement 3: UTI signs and symptoms identified by NLP.**

| **Category** | **Sign or symptom** | **Synonyms included (not exhaustive) and notes** |
| --- | --- | --- |
| Symptoms suggestive of UTI | Dysuria | Painful urination; discomfort with urination; burning with urination. Note: malodorous urine *not* included[43](https://onlinelibrary-wiley-com.yale.idm.oclc.org/doi/10.1111/acem.14883#acem14883-bib-0043) |
|  | Hematuria | Bloody urine. Note: dark urine *not* included. |
|  | Urinary frequency | Frequent urination. |
|  | Urinary urgency | Need to urinate; urge to urinate. |
|  | Urinary retention | Inability to urinate; difficulty urinating. Note: decreased urination *not* included. |
|  | Urinary incontinence | Urinating on self. |
|  | Abdominal pain | Abdominal discomfort, suprapubic pain. |
|  | Flank pain | Side pain. |
|  | Low back pain | Lumbar pain. |
|  | Pelvic pain | Groin pain. |
|  | Back pain | Note: thoracic back pain or back pain with unspecified region. |
| Systemic symptoms potentially related to UTI | Fever | Specific elevated temperatures > 38°C. Note: chills *not* included. |
|  | Fatigue | Malaise, lethargy, generalized weakness. |
|  | Altered mental status | Confusion, encephalopathy. |
| Exam findings suggestive of UTI | Suprapubic tenderness | Tenderness over bladder. |
|  | Costovertebral angle tenderness | CVA tenderness; flank tenderness. |
|  | Abdominal tenderness | Note: Any abdominal tenderness not in above regions or with unspecified region. |

**Supplement 4:** Model feature completeness and missingness.

| **Feature (urinalysis component)** | **Present (%)** | **Missing (%)** |
| --- | --- | --- |
| Bacteria | 89,063 (59.6) | 60,386 (40.4) |
| Blood | 148,517 (99.4) | 932 (0.6) |
| Epithelial cells | 27,908 (18.7) | 121541 (81.3) |
| Leukocyte esterase | 148,537 (99.4) | 912 (0.6) |
| Nitrite | 148,532 (99.4) | 917 (0.6) |
| White blood cells | 77,509 (51.9) | 71940 (48.1) |
| Glucose | 148,534 (99.4) | 915 (0.6) |
| Ketones | 148,533 (99.4) | 916 (0.6) |
| Protein | 148,533 (99.4) | 916 (0.6) |

**Supplement 5:** Model and physician performance (overdiagnosis and underdiagnosis) according to the *liberal* UTI definition, requiring culture positivity alone. Model predictions are shown at a diagnostic threshold of 28%. CV for accuracy and DOR are displayed below.


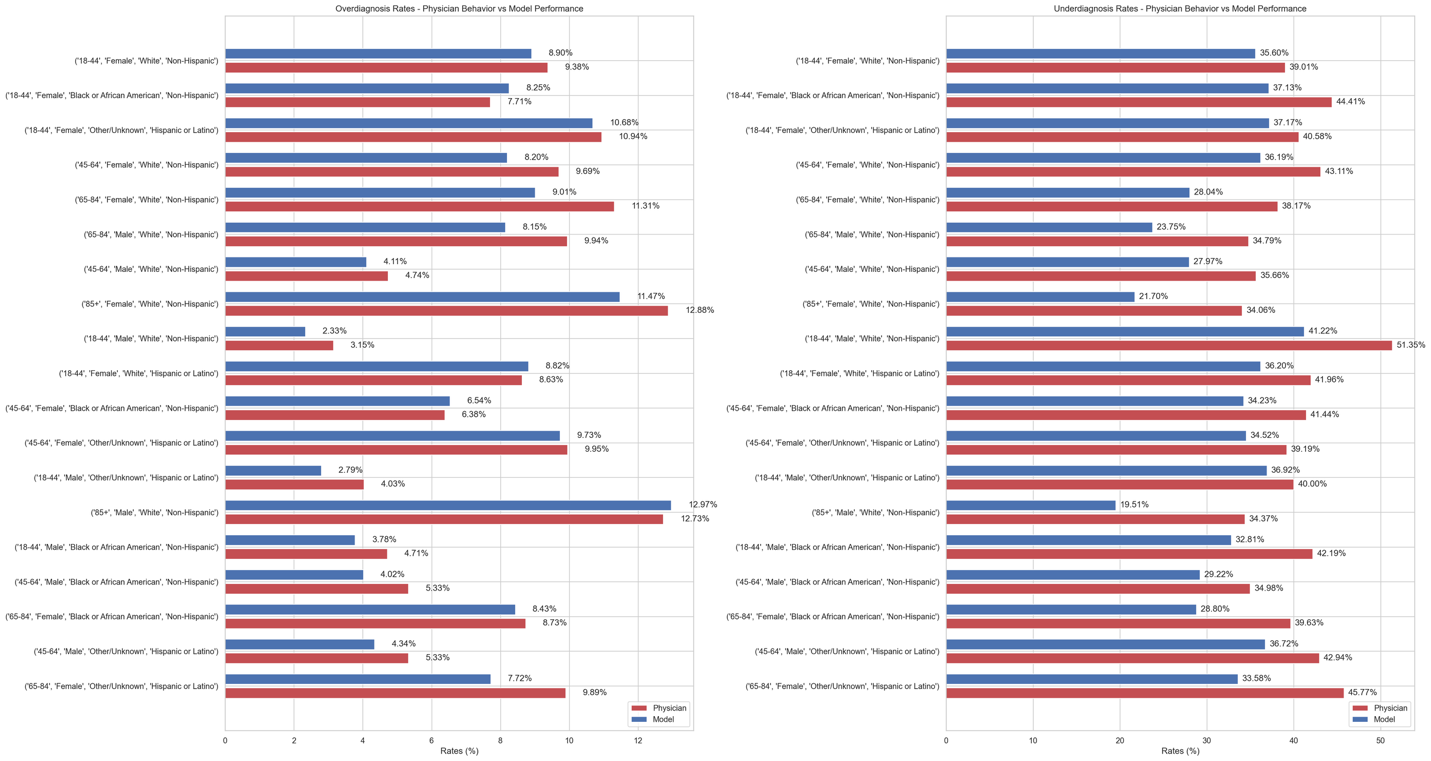


|  | **CV for accuracy (95% CI)** | **CV for DOR (95% CI)** |
| --- | --- | --- |
| **Model** | **0.044 (0 to 0.36)** | **0.49 (0.15 to 0.84)** |
| **Physicians** | **0.051 (0 to 0.37)** | **0.44 (0.11 to 0.77)** |

**Supplement 6:** Model and physician performance (overdiagnosis and underdiagnosis) according to the *strict* UTI definition. Model predictions are shown at a diagnostic threshold of 42.3%. CV for accuracy and DOR are displayed below.


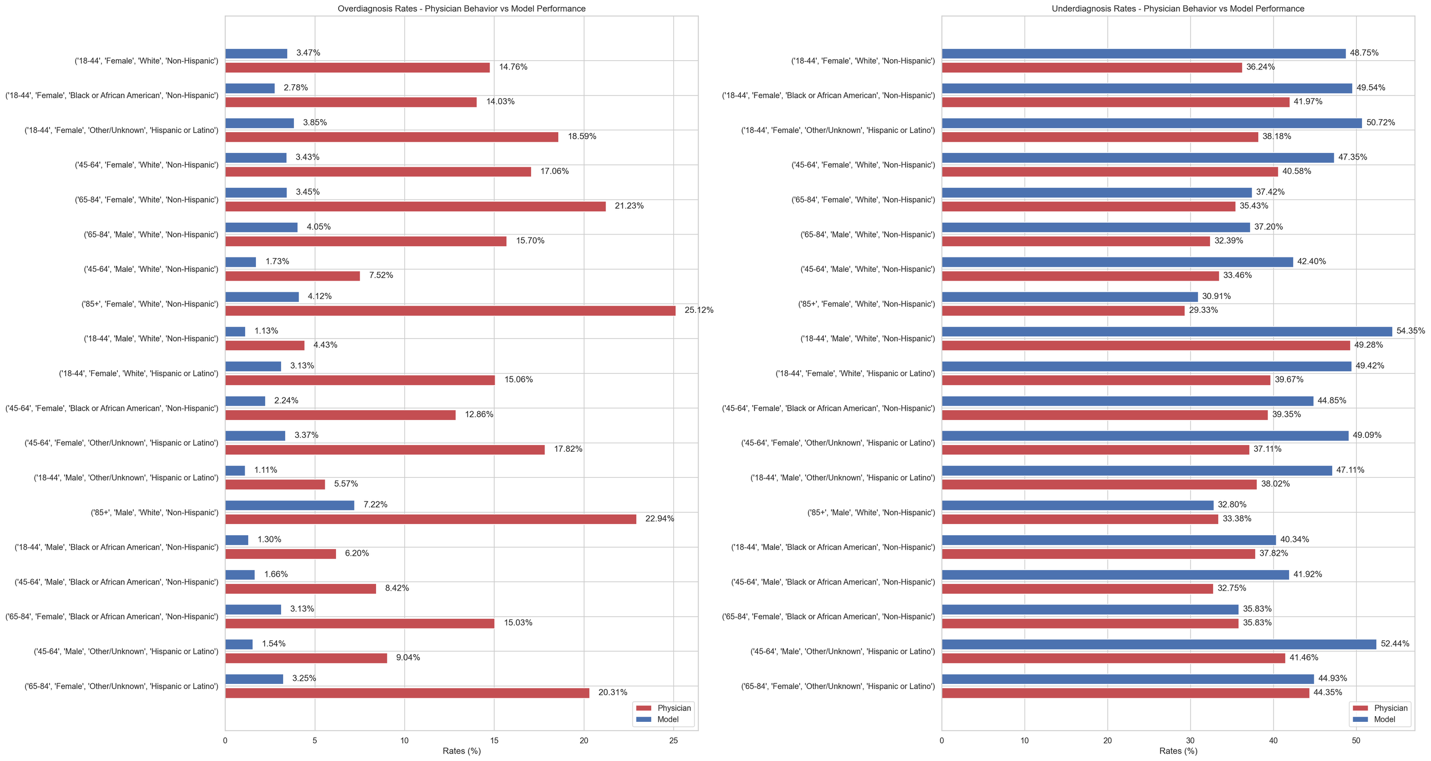


|  | **CV for accuracy (95% CI)** | **CV for DOR (95% CI)** |
| --- | --- | --- |
| **Model** | **0.034 (0 to 0.35)** | **0.50 (0.16 to 0.84)** |
| **Physicians** | **0.080 (0 to 0.40)** | **0.33 (0 to 0.66)** |

**Supplement 7:** Model and physician performance (overdiagnosis and underdiagnosis) according to the *strict* UTI definition, using the “optimal” diagnostic threshold of 15.0%. CV for accuracy and DOR are displayed below.


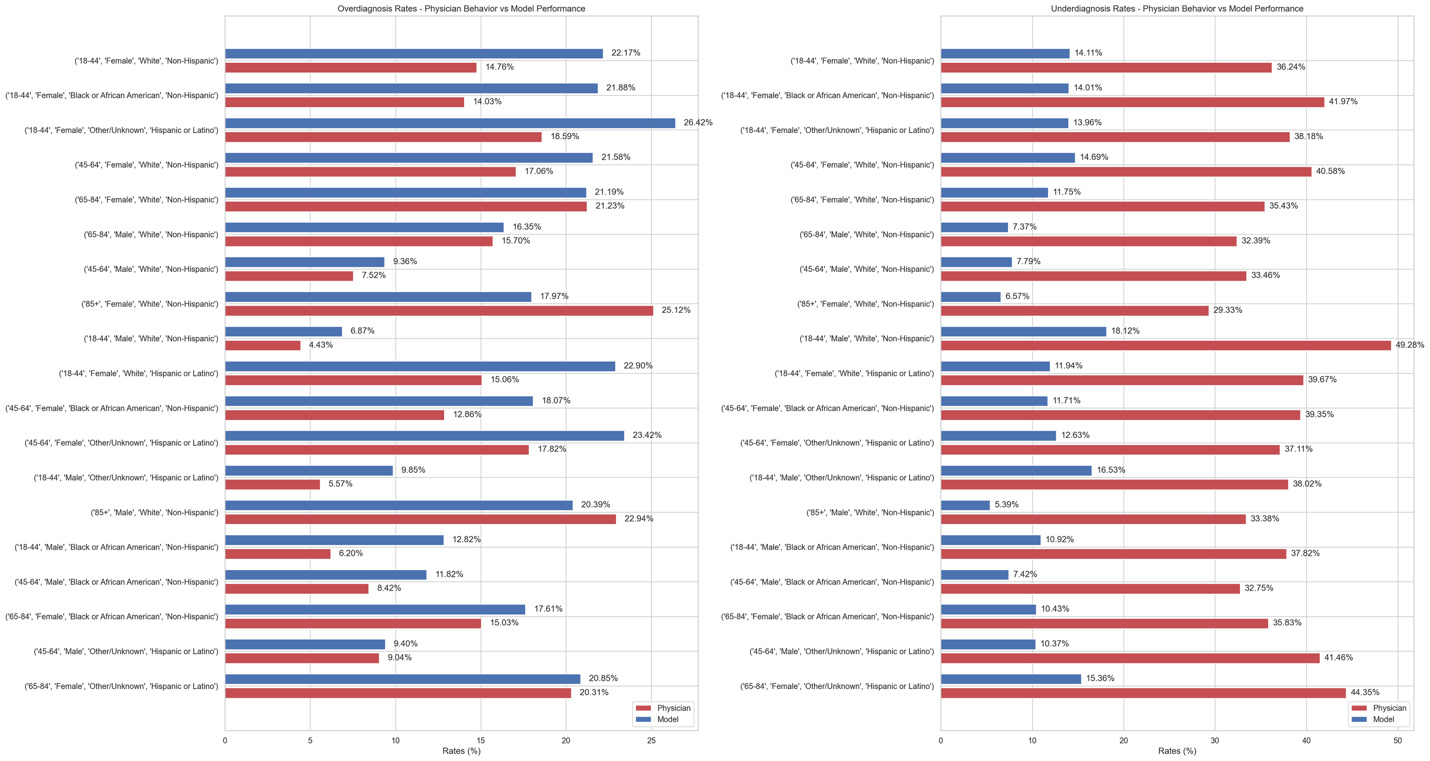


|  | **CV for accuracy (95% CI)** | **CV for DOR (95% CI)** |
| --- | --- | --- |
| **Model** | **0.064 (0 to 0.38)** | **0.61 (0.27 to 0.96)** |
| **Physicians** | **0.080 (0 to 0.40)** | **0.33 (0 to 0.66)** |

**Supplement 8:** Comparison of rates of physician UTI diagnosis (bars to left) and culture positivity (bars to right) for each decile of model-predicted likelihood of culture positivity.


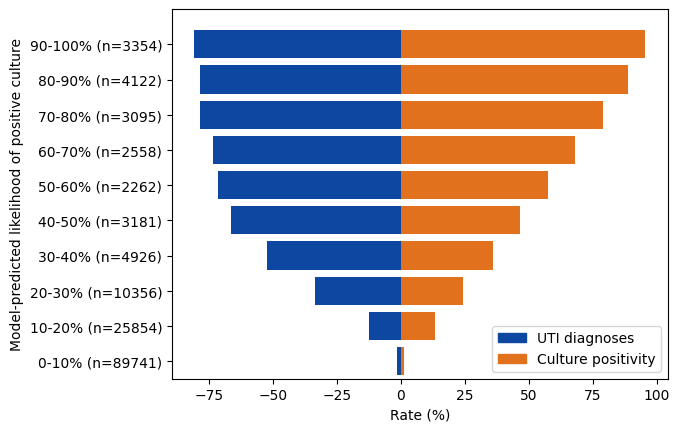

Supplement: Multimedia Appendix 1 [file ai-v5-e91148-s001.docx]
